# Direct and Indirect Costs of Diabetes in Brazil in 2016

**DOI:** 10.5334/aogh.3000

**Published:** 2022-03-03

**Authors:** Paula Pereda, Vanessa Boarati, Bruna Guidetti, Ana Clara Duran

**Affiliations:** 1 Department of Economics, School of Business and Economics, University of Sao Paulo, BR; 2 Center for Food Studies and Research (NEPA), University of Campinas, BR; 3 Center for Epidemiological Studies in Nutrition and Health (NUPENS), University of Sao Paulo, Av. Albert Einstein, 291 Campinas, SP, Brazil

## Abstract

**Background:**

The global economic burden of *Diabetes mellitus* (DM) is expected to reach US$ 745 billion in 2030. The growing prevalence of the disease, mainly type 2 diabetes, is the result of population aging, nutritional transition, which include growing rates of obesity and consumption of foods high in sugar and fat. Brazil is the fourth country in the number of patients with diabetes globally and follows the global trends, with a continuous increase in prevalence.

**Objectives:**

To estimate the economic burden of DM in Brazil, including all direct and indirect costs.

**Methods:**

We used a cost-of-illness approach to calculate the total economic burden of DM. We used official healthcare-related statistics referring to 2016.

**Findings:**

We estimated the Brazilian economic burden to reach US$ 2.15 billion in 2016, of which 70.6% are indirect costs related to premature deaths, absenteeism, and early retirement. We estimate that if the rate of growth of diabetes prevalence remains in Brazil, direct and indirect costs of diabetes will more than double by 2030 (an increase of 133.4% or 6.2% per year).

**Conclusion:**

Our results are in accordance with the literature that shows that indirect costs are more relevant in low- and middle-income countries due to poorer access to health care, which result in higher mortality rates from non-communicable diseases. However, due to the potentially underestimated prevalence of diabetes in Brazil and the lack of access to nationwide private healthcare costs, we estimate costs of diabetes in Brazil to be higher than the conservative results we found. The onset of the COVID-19 pandemic is likely to result in even greater costs than what we estimated.

## 1 Introduction

*Diabetes mellitus* (DM) is one of the most prevalent non-communicable diseases (NCDs) in the world, and the third highest risk factor for premature mortality after high blood pressure and tobacco use [10]. The global prevalence of DM increased from 108 million adults (4.7% of the total population) in 1980 to 415 million in 2015 (8.8% of the total population) [33,35]. The rapid growth of DM prevalence, especially of type 2 (TDM2), is mainly caused by population aging, nutritional transition (unhealthy eating and lifestyles that are associated with growing obesity levels), economic growth and unplanned urbanization [11,34]. In the context of the recent COVID-19 pandemic, caused by the SARS-CoV-2 virus, evidence suggest DM is an underlying comorbidity that increases the chances of mortality and developing the severe form of the disease, thus requiring intensive care unit (ICU) admission [15,25,27,28].

In this sense, DM is associated with growing costs to health systems (disease and co-morbidity treatment), and therefore to society as a whole [3]. In 2010, the global economic burden of DM was estimated to reach US$ 500 billion and was forecasted to reach US$ 745 billion by 2030 [10]. The economic burden of DM includes direct medical costs (medicines, health services, emergency rooms, and hospitalization expenses), direct non-medical costs (patient transportation) and indirect costs (productivity effects, early retirement, absenteeism, premature mortality and worsen quality of life) [4,12].

Whether direct or indirect costs are the main drivers of the economic burden of DM differs by economic development and access to health care. In developed countries, the direct costs tend to be higher than non-medical and indirect costs [19,43] – and in settings without universal access to healthcare, such as the USA, direct costs represent 72.5% of total costs (US$ 237 billion out of US$ 327 billion) [5]. In low- and middle-income countries, the indirect costs of DM are more relevant, primarily because of higher DM mortality rates [1,21,29] and weaker health systems (insufficient and/or inefficient) [8,9,16,29,32].

Despite the efforts of the Brazilian public health care system (SUS), Brazil is the fourth country in the world in number of adults with DM (7.7% of Brazilians 18 or older in 2019) [6,18,22]. The literature also shows that DM prevalence might be underestimated in the country [17,30]. In a cohort of university public servants 50% of the cases were previously undiagnosed [41]. In 2015, 7% of all disabilities, with an annual loss of 4,049,510 Disability-Adjusted Life Years, could be attributed to DM in Brazil [20], and women were more affected than men [17].

Diabetes-related health expenditures with hospitalizations in Brazil were estimated to be around US$265 million in 2014 [38]. In 2007, the DM total annual economic burden in the Brazilian public health system was US$2,108 per patient, of which 63.3% were direct costs and 36.7% indirect costs [7]. Another study estimated DM costs in 2010 for the city of São Paulo. The authors found that DM costs US$1,844 per patient (55% direct costs and 45% indirect costs) [13,14]. A recent study, applying the attributable risk method, estimated an average total cost of US$845 per patient [37]. Despite the fact that a few studies have attempted to calculate the economic burden of DM in Brazil, they do not consider all the opportunity costs involved in treatment and management of the disease or attempted to estimate the contribution of private healthcare system costs in the country, which cover around 23% of the population [2]. Considering the lack of comprehensive nationwide estimates, we intend to fill this gap by estimating the direct and indirect economic burden of DM (cost-of-illness method) in Brazil using a diverse set of datasets and official statistics.

## 2 Materials and Methods

### 2.1 Study Design

We employed a prevalence-based approach that combines the most recent nationwide estimates of diabetes prevalence, epidemiological data, health care cost, and economic data using a cost-of-illness method.^1^

### 2.2 Data sources and analysis

#### 2.2.1 Population size

We estimated DM prevalence and total population with DM using the most recent National Health Survey, conducted in 2013, which provides nationwide self-reported estimates of DM for the Brazilian adult population stratified by sex (men, women) and age groups (<18, 18–34, 35–44, 45–54, 55–64, 65–74 and 75+) [26].

To estimate diabetes prevalence in 2016, we scaled the estimates based on population growth between 2013 and 2016 by sex and age groups using population projection estimates conducted by the Brazilian National Bureau of Statistics for 2016 [24].

#### 2.2.2 Direct costs

The method we used to measure the economic burden of DM in Brazil is similar to previous studies on the economic burden of DM. Unless otherwise specified, we defined an event caused by DM when the primary cause of hospitalization was classified between E10 and E14 of the International Classification of Diseases (ICD 10). We excluded DM acquired during pregnancy [5,11].

To calculate all the economic costs associated with DM, we accounted for the relationship between DM and the incidence of other diseases. We reviewed the medical literature involving the increase in risks of some conditions due to DM [4] to understand this relation. The list of diagnoses included in this second group was adapted from a study on hospitalizations cost in Brazil [38] and included the following conditions: microvascular complications; macrovascular complications; respiratory and urinary tract infections; neurological; renal and eye diseases; and selected cancers (breast, liver, colorectal, endometrium, and pancreas neoplasms).

The concept of Population Attributable Risk is used to measure the relationship between DM and other diseases to assess the important second-order effect of DM prevalence. The Population Attributable Risk indicates the proportion of cases that would not occur if DM was absent. This concept depends on the prevalence of the risk factor and the risk of exposure (Relative Risk, RR) of the disease:

1 shar{e_g} = \frac{{{P_g}x\left({R{R_g} - 1} \right)}}{{\left[ {1 + {P_g}x\left({R{R_g} - 1} \right)} \right]}}

The fraction is calculated using DM prevalence rate (P) and the relative ratio risk (RR) for each chronic complication of DM [37]. The subscript g refers to age groups. The idea is to consider all the health issues from DM along with diseases related to DM. In this sense, the Population Attributable Risk determines the share of health problems due to diseases commonly classified as morbidity of diabetes.

We considered only adults older than 35 years old to calculate the costs associated with DM-related diseases because information on the relative risk of such conditions associated with DM is scarce for younger individuals due to the lower prevalence of DM among this group. The parameters are calculated based on the National Health Survey for 2013 (PNS-2013), stratified by gender and age groups.

The economic costs of DM were calculated using the cost-of-illness (COI) approach, adding the medical and non-medical costs of DM and other diseases associated with DM for Brazil. Direct costs refer to health sector costs for disease prevention, diagnosis, and treatment. In this paper, the following expenditures are included: expenses for hospital care (hospital services and physicians/other healthcare professionals), medical services (outpatient), and pharmaceutical expenses. All the costs were disaggregated by gender (men and women) and age (<18, 18–34, 35–44, 45–54, 55–64, 65–74 and 75+). Costs related to hospitalization and ambulatory procedures were also divided into costs incurred by the Brazilian public health system (SUS) and private costs, which include both out-of-pocket and insured medical services.

*Hospitalization cost*: Hospitalizations were categorized into two groups: (1) those in which the main diagnosis was reported as DM and coded as ICD10 E10 to E14; (2) those in which the main diagnosis was reported as any chronic complication of DM and related diseases, including infectious and neoplastic diseases for which DM is considered to be an important risk factor.

We collected hospitalization data from the National Public Healthcare Hospitalization System (SIHSUS) for the public health system and from the Communication System of Hospital and Ambulatory Information (CIHA) for private health system costs. Both datasets are generated by DATASUS (Information Technology Department of the Public Health Care System), and provide data on the hospitalization date, length of stay, days in an intensive care unit (ICU), gender, age, among others. It is noteworthy that only SIHSUS presents the average costs per hospitalization.

Costs in SIHSUS were disaggregated into expenses for healthcare professionals and materials. These data also provided the cost of days spent in ICUs. Therefore, we use SIHSUS costs as a lower-bound approximation for private average costs, as public prices of services and medication are lower than market prices in Brazil.

*Ambulatory Cost*: We collected data from two sources: (i) the Ambulatory Information System (SIASUS), which considers all outpatient procedures paid by the public health system; and (ii) the Private Healthcare Ambulatory Information System (CIHA), which includes costs associated with the private health system. Considering CIHA does not provide cost information, we used the average cost per utilization stratified by age and gender provided by the Ambulatory Information System (SIASUS) to estimate the costs of the procedures and utilization in the private health system.

Ambulatory costs were also categorized into two groups: (1) those in which the main diagnosis was reported as DM and coded as ICD10 E10 to E14; (2) those in which the main diagnosis was reported as any chronic complication of DM and related diseases, including infectious and neoplastic diseases for which DM is considered to be an important risk factor.

*Medication costs*: We collected data from the Popular Pharmacy program (PP). This Brazilian government program provides commonly used drugs at a reduced price or free, including several free diabetes drugs. The entire Brazilian population is eligible for the program, but monthly purchases are capped to the amount to be used by the individual per month. The medication can be purchased at any accredited private pharmacy or program pharmacy. Microdata for 2016 were obtained by invoking the Brazilian Information Access Law. The pharmaceuticals include human insulin and oral medicines [40].

*Other out-of-pocket costs for materials*: We collected data from the last available National Household Budget Survey (POF) to calculate direct costs related to materials associated with capillary blood glucose testing. No other material costs related to diabetes care were available at POF. POF is conducted by the Brazilian Institute of Geography and Statistics (IBGE). Since the last data available refer to January 2009, we inflated the costs to 2016 prices using the National Consumer Price Index (IPCA-e) between 02/2009 and 12/2016. We did not use the cost of the Popular Pharmacy Program to measure expenditures with materials to avoid double-counting, since it was still incipient when the last household survey was conducted [23].

#### 2.2.3 Indirect costs

We considered the following indirect costs in the analysis.

*Absenteeism*: Denotes the lost productivity due to sickness requiring absence from work. We used the number of workdays missed (WDM) per year per person due to the disease. This measure corresponds to the number of workdays missed (WDM) multiplied by the daily average minimum wage. In our data, absenteeism is a direct implication of hospitalization, even for children and the elderly, since they may require the accompaniment of some adult (possibly of working age). Children and adolescents up to 18 years old and elderly (above 60 years) have the right to have one companion in the hospital while hospitalized [12,13].

We used the minimum monthly salary of US$441.10 (in 2016) to evaluate a missed day for people aged between 18 and 64. For the younger and older than 65, we attributed the average salary of those aged between 18 and 64 as an opportunity cost.

*Premature death (PD)*: Denotes the economic losses due to labor decreases as a result of terminal conditions. We calculated PD losses from the present value of the labor market outcomes lost prior to retirement based on age. According to the Brazilian pension system (in 2016), private-sector employees are entitled to retirement if they meet one of two conditions:(i) retirement based on age 65 for men and 60 for women with a minimum length of the contribution of at least 15 years, and (ii) retirement based on length of contribution – 35 years for men and 30 years for women:

2 P{D_{ij}} = \mathop \sum \limits_{t = 1}^T \frac{{{N_{ij}}\omega }}{{{{\left({1 + r} \right)}^t}}}

In which *N_ij_* is the number of deaths per year of individuals of gender *i* and age *j, w* is the minimum salary established in January 2016, *r* is the real interest rate in Brazil (average between 2012 and 2016). The rate was calculated by subtracting the average Brazilian benchmark interest rate (SELIC rate, disclosed by the Brazilian Central Bank) by the accumulated inflation for the year (National Consumer Price Index – IPCA).

The *T* = (*L_ij_ –AR_i_*) is the difference between the age of mortality, *L_ij_*, and age of retirement of the gender, *AR_i_*, for those above the minimum legal age (18 years old).

To calculate YLL, we used data on premature deaths available at the Mortality Information System (SIM) in 2016. This publicly available dataset is disclosed annually and contains information on all deaths in Brazil. We attributed a minimum value to the lost years of life in productivity. In this calculation, we considered deaths of which DM was the cause. We understand that our decision to consider deaths attributable to DM potentially underestimates the number of deaths due to diabetes. When admitted to in-patient or out-patient care, patients may either fail to mention they have diabetes or may not know they have the disease [30]. Also, healthcare workers may fail to include diabetes as a cause of death [30]. Moreover, the mortality risk among people with diabetes is higher than the mortality risk for the general population [20]. We restricted the data to 60 years for women and 65 years for men, as those are the legal ages for retirement. We also excluded deaths of those below 18 years old. As mentioned, we used minimum wage to represent the productivity loss in the labor market and the Brazilian average real interest rate to calculate net present values for 2016.

*Disability or early retirement (DIS)*: Represents the value of interruption of employment due to sickness related to DM. The cost is estimated using the average amount paid by the government to new retirees and sickness aid due to DM in 2015. The number of beneficiaries is determined as the number of people retiring early due to disabilities related to severe diabetes.

Finally, to calculate the cost due to early retirement related to DM complications, we proxied the number of beneficiaries by people under the legal age of retirement who have DM in the most recent Brazilian National Health Survey (PNS 2013). We considered data of new disability benefits granted, which include retirement for disability due to injury and sickness. We have also restricted the data to benefits related to DM according to ICD-10. As the most recent data are for 2015, we inflated the values to 2016 using the price consumer index and the real interest rate. We only considered men until 65 years and women until 60 years, as these are the legal ages for retirement in Brazil.

#### 2.2.4. Economic burden projection

We projected future direct and indirect costs of diabetes to 2030 based on two scenarios. The first is a conservative scenario in which we considered that the DM prevalence would remain the same as it was found in the 2013 Brazilian National Health Survey (6.2%). In the second scenario, which we judge to be more realistic, we considered that the prevalence of diabetes in Brazil will grow from 2016 to 2030 at the same annual rate it grew between 2003 and 2013 (calculated using the PNAD 2003 and PNS 2013 surveys). In both situations, we consider the prevalence by sex and age groups. We did not include people under 18 years old since data on diabetes prevalence for children and adolescents are not available in either survey. In this scenario, the diabetes prevalence in Brazil is a weighted average of the groups’ prevalence. On the cost side, we used our estimate of diabetes cost, maintaining it constant over time.

Finally, the number of people with diabetes was calculated by multiplying the estimated prevalence of diabetes in 2030 by the projected population in 2030 according to IBGE. The future cost is given by the product of the cost per person calculated in this study and the estimated number of diabetics in 2030 across age groups.

### 2.3 Sensitivity analysis

We ran sensitivity analysis to try to estimate the costs of the private health care that covers 23% of the Brazilian population by using data from hospitalization provided by a health care system in a Brazilian state located in the Southeast of the country [2].

We use hospitalization prices from Diagnosis Related Group (DRG-Brazil) – a dataset composed of data from more than 200 hospitals in the country. In 2016, this dataset included 4,909 patients that were hospitalized due to DM or complications of DM [31].

## 3 Results

We estimated total costs of US$2,153.05 million: US$633.03 million (29.4%) in direct costs and US$1,520.02 million (70.6%) in indirect costs in 2016.

### 3.1 Direct costs

In 2016, the total direct costs related to DM were US$633 million. ***Table 1*** shows all components of the direct costs. When it comes to hospitalizations, we estimated total expenses to be US$232.8 million (81.4% of the total). When accounting for DM expenses and related conditions, women’s hospitalization expenses were US$108.6 million (46.7%), while the expense for men were US$124.1 million (53.3%). Direct costs of hospitalizations, only related to DM, represented US$50.2 million for the public health system. On the other hand, DM-related conditions summed US$139.7 million in the public health system.

**Table 1:** Direct costs estimates, in 2016 million US$.


	TOTAL	DM AS MAIN CAUSE	MORBIDITIES RELATED TO DM

*Hospitalizations*	232.8	58.1	174.7

Women	108.6	30.4	78.2

Men	124.1	27.7	96.4

Public	189.8	50.2	139.7

Private	43.0	8.0	35.0

*Ambulatory*	86.0	3.9	82.1

Women	49.0	1.9	47.1

Men	37.0	1.9	35.1

Public	82.5	3.5	79.0

Private	3.5	0.4	3.1

*Popular Pharmacy*	304.2		

*Out-of-Pocket Expenses*	10.0		

**Total**	**633.0**		


Regarding outpatient costs, we calculated a total of US$ 86 million in 2016 (the public health system covered 95% of those costs). Most of costs were related to women (57% of such costs; US$49 million), while men’s costs totaled US$37 million (43%). The total costs of outpatient care attributed to DM (US$ 82.1 million) were 21 times higher than total DM outpatient costs when DM was the main cause of admission (US$ 3.9 million).

The same results of ***Table 1*** are also disaggregated by age groups in ***Table 2***. Costs for DM as the main cause was calculated in seven different age groups, while costs attributed to DM were calculated by five age groups due to the availability of information on relative risks. In both cases, we observed some heterogeneity. The age groups 55–64, 65–74, and 75 years and older are responsible for 80.7% of total hospitalization costs and 86.0% of ambulatory costs.

**Table 2:** Hospitalization and ambulatory costs by age groups, in 2016 million US$.


	DM AS MAIN CAUSE		ATTRIBUTED TO DM		TOTAL	

*Hospitalizations*	58.1		174.7		232.8	

below 18 years old	3.5	6.0%			**3.5**	**1.5%**

18–34 years old	7.7	13.3%			**7.7**	**3.3%**

35–44 years old	6.3	10.8%	3.8	2.2%	**10.1**	**4.3%**

45–54 years old	7.6	13.1%	16.2	9.3%	**23.8**	**10.2%**

55–64 years old	11.5	19.7%	48.7	27.9%	**60.2**	**25.8%**

65–74 years old	11.6	19.9%	60.6	34.7%	**72.2**	**31.0%**

75 years old and above	10.0	17.3%	45.4	26.0%	**55.4**	**23.8%**

*Ambulatory*	3.9		82.1		86.0	

below 18 years old	0.04	1%			**0.04**	**0.05%**

18–34 years old	0.2	4%			**0.2**	**0.2%**

35–44 years old	0.3	7%	2.1	2.6%	**2.4**	**2.8%**

45–54 years old	0.7	19%	8.1	9.9%	**8.8**	**10.3%**

55–64 years old	1.3	34%	23.5	28.6%	**24.8**	**28.8%**

65–74 years old	0.9	24%	31.4	38.3%	**32.3**	**37.6%**

75 years old and above	0.4	12%	17.0	20.7%	**17.4**	**20.3%**


Finally, the total direct costs include drugs (US$304 million) and materials (US$10 million). Due to data limitations, these total direct costs could not be divided by gender or age group.

### 3.2 Indirect costs

Total indirect costs are calculated by the sum of economic losses due to absenteeism, premature deaths, and early retirement caused by DM and its complications. In 2016, we estimated that they represent US$1.52 billion.

We use hospitalization data to calculate absenteeism (***Table 3***), which has a small share of total indirect costs (2.6%), amounting to US$39.8 million (US$20.1 million for men, 50.4%, and US$19.1 million, for women, 49.6%). The results are homogeneous by gender but not by age group. The groups above 55 years old are responsible for more than 75% of total absenteeism costs.

**Table 3:** Indirect costs estimates, absenteeism, in 2016 US$ million and % of total.


	TOTAL	DM AS MAIN CAUSE			ATTRIBUTED TO DM	
	
		WOMEN	MEN		WOMEN	MEN

*Absenteeism (in million)*	39.80	10.20	9.59		9.52	10.49

below 18 years old	2.53%	5.69%	4.44%			

18–34 years old	4.62%	13.17%	5.18%			

35–44 years old	5.37%	9.69%	7.63%		2.02%	2.15%

45–54 years old	11.43%	11.92%	17.34%		7.70%	8.95%

55–64 years old	23.42%	18.91%	26.12%		21.48%	27.12%

75 years old and above	27.29%	20.48%	23.29%		30.21%	34.92%


The indirect costs due to premature deaths due to DM (***Table 4***) were US$1.18 billion (77.9% of total indirect costs): US$409 million for women and US$ 775 million for men. We did not estimate the indirect costs of premature death for chronic complications of DM and related diseases.

**Table 4:** Indirect costs estimates, premature death, in 2016 US$ million and % of total.


	TOTAL	DM AS MAIN CAUSE	

		WOMEN	MEN

*Premature Death (in million)*	1,183	409	775

18–34 years old	14.38%	20.07%	11.38%

35–44 years old	20.10%	25.29%	17.37%

45–54 years old	38.53%	40.98%	37.24%

55–64 years old	26.99%	13.66%	34.02%


Likewise, the total indirect costs of early retirement were estimated only for those whose main diagnosis is DM due to lack of more information. The total costs of early retirement were US$296.7 million (19.5%), of which US$186.4 million (7.3%) is for women and US$110.4 (12.3%) for men.

### 3.3 Economic burden of DM per patient

In 2016, 9,631,664 Brazilians over 18 were estimated to be diagnosed with diabetes (prevalence rate of 6.4% according to the National Health Survey for 2013). Therefore, ***Table 5*** shows that the total cost per patient diagnosed in Brazil with DM is US$223.54: US$65.72 of direct costs (or 29.40% of the total), and US$157.81 of indirect costs (or 70.60% of the total).

**Table 5:** Economic burden of DM per patient and *per capita*, in 2016 values.


	DIRECT COSTS	INDIRECT COSTS	TOTAL

Total Economic Burden (in million US$)	633	1,520	2,153

% of the total	29.40%	70.60%	

Economic Burden *per capita* (in US$)	3.09	7.41	10.49

Economic Burden per patient (in US$)	65.72	157.81	223.54


When we calculated those costs for the Brazilian population, the total costs per capita represented US$10.49: US$3.09 as direct costs per capita, and US$7.41 as indirect costs.

### 3.4 Projection of the economic burden in 2030

In order to project our estimates to 2030, we considered two different scenarios, as ***Table 6*** shows. In the first scenario, we considered that the prevalence of DM would remain the same as the latest available estimate for 2013 and would only increase as the population age. In this case, the average prevalence rate would increase from 6.4% in 2016 to 7.9% in 2030. The total economic burden would increase from US$ 2,15 billion to US$ 3.06 billion in 2030 (a real increase of 42.2%, or 2.6% per year). In the second scenario we considered, which we believe to be more realistic, the prevalence rate would increase to 13.0% in 2030. In this scenario, the total economic burden would increase to US$ 5.03 billion, an increase of 133.4% or 6.2% a year.

**Table 6:** Total economic burden (EB) of DM, current (2016) and projected (2030), in 2016 million US$.


	DM PREVALENCE	DM PATIENTS	TOTAL EB	% OF EB	% ANNUAL EB

	%	N	U$ MILLION	%	%

2016 values	6.42%	9,631,664	2,153.05		

2030 optimist^[1]^	7.90%	13,695,390	3,061.45	42.19%	2.55%

2030 realist^[2]^	12.96%	22,479,515	5,025.05	133.39%	6.24%


*Notes*: [1] We consider that DM prevalence changes only due to population aging. [2] We consider that DM prevalence changes both with population aging and at the same pace it increased from 2003 and 2013.

### 3.5 Sensitivity analysis

We ran a sensitivity analysis using hospitalization prices provided by DRG-Brazil. Total costs of hospitalization using DRG prices are US$422.5 million. When using such prices, the total diabetes costs estimated reached US$2,342.7 million: US$822.7 million in direct costs (35.1%) and US$1,520.01 million in indirect costs (64.9%) in 2016.

## 4 Discussion

Using conservative estimates, we calculated that the Brazilian burden of diabetes in 2016 was US$2.15 billion, or 0.12% of the country’s GDP. Most of the burden is attributed to indirect costs (70.6%), as previous studies in Brazil indicate [7]. Our results are consistent with the literature in middle- and low-income countries, in which the health system weaknesses increase the participation of mortality and opportunity costs of the disease. However, considering a potentially underestimated prevalence of diabetes in Brazil of at least 50%, we estimate our costs to be highly underestimated [41]. The recent COVID-19 pandemic may increase direct and indirect costs of DM in Brazil, considering DM has been associated with severe cases of COVID-19, and thus admission to intensive care units [28]. A published meta-analysis that included 16,003 patients mostly from mainland China, but also France and the United States, found an increased odds ratio of severe cases of COVID-19 and a two-fold increase in mortality in patients diagnosed with COVID-19 with a previous diagnosis of DM as compared with patients without a DM diagnosis [27].

Another limitation usually referred to in the literature [41] includes the lack of data on the cost incurred by the private healthcare system in Brazil, which covers around 23% of the population. Using data from a provider located in a state in the Southeast of Brazil, we found hospitalization costs 30% higher than those found for the public hospitalization system. We contribute to the literature on DM costs in Brazil by combining the best publicly available data sources with information on a single but large private healthcare provider to assess a comprehensive set of direct and indirect costs of DM. Many of the previously published studies on costs of DM in Brazil did not consider detailed assumptions about the cost estimates that have potentially led to important underestimations in the economic cost of DM in Brazil, nor were they able to include different opportunity costs involved in diabetes treatment and prevalence [7,13,14].

We were also able to disaggregate the results by gender and age group. In this case, we assessed the heterogeneity of the results, including hospitalization and ambulatory costs. For direct costs, DM is the main diagnosis across seven of the age and gender groups and costs attributed to DM in five of these groups. The elderly individuals (above 65 years) are responsible for 40% to 70% of total hospitalization and ambulatory costs. This result is similar to the literature, which also shows that individuals older than 65 are responsible for a much larger proportion of hospital resources (hospitalization and ambulatory) [4,37].

The analysis of gender costs provides interesting results. We find that ambulatory costs are more relevant among women, probably due to the greater attention that women give to their health than men [39,42]. The same argument explains the larger premature death costs we find for men.

Our conservative analyses also did not consider costs associated with the management of renal diseases alone, which can be responsible for a large share of the costs associated with the complications of diabetes [41].

One important limitation of all the economic burden of DM analysis carried out in Brazil is the lack of price information of the medical services provided by the private sector. Previous studies assume that the service prices are the same as those paid by the government, which is a very conservative hypothesis that underestimates direct cost estimates. In this sense, we ran a sensitivity analysis using price estimates provided by a private healthcare provider located in the State of Minas Gerais, Brazil. The average price of medical services of this private system is 30% higher than that paid by the public health system. Despite our inherent limitation associated with using data from a single provider, our results show that the current estimates of direct costs of diabetes in Brazil in the literature – along with ours – are underestimated. This provider, however, serves 200 hospitals and covers 1.5 million lives.

Another limitation of our analysis includes the lack of other negative externalities of DM in Brazil. Negative externalities might occur when the health system does not absorb the increased demand for DM patients, generating spillovers for other patients.

Finally, we could not include judicialization costs of diabetes treatment in our analyses. The list of medicines available at the public healthcare system in Brazil is limited. For instance, insulin analogs and new oral drugs are not widely for the population. Because patients can file lawsuits to assert their constitutional right to health and thus request access to medicines and treatments not covered by the national public healthcare system at no cost. Although nationwide data are not available, findings from the state of S˜ão Paulo – the most populated and affluent in the country – show that 25% of all lawsuits of this type involve DM-related drugs [36].

## 5 Conclusion

We calculated the total economic burden of *Diabetes mellitus* in Brazil using a cost-of-illness approach using several data sources. Our conservative analyses show that the cost of the disease represented at least 0.12% of the country’s GDP in 2016, with a potential to more than double in the next 14 years. The indirect costs accounted for the highest proportion of total DM costs. The undiagnosed cases of diabetes, the unidentified deaths by diabetes, and our limitations in accessing the unit and per capita cost associated with the complementary private healthcare system that cover a fifth to a quarter of the Brazilian population are the reasons why our estimates are conservative and that the total DM economic burden in Brazil is even higher that what we calculated.

## Additional Files

The additional files for this article can be found as follows:

10.5334/aogh.3000.s1 Appendix A. Costs attributed to DM – Hospitalization and ambulatory.

10.5334/aogh.3000.s2 Appendix B. Direct Costs – DM and Morbidities.

## Data Accessibility Statement

The majority of the data used are open sourced. Part of the complementary data is the Diagnosis Related Group (DRG) dataset. These data are not publicly available and were kindly provided by Ana Claudia Abreu, Tania Grillo, and Renato Couto at the Federal University of Minas Gerais.

## Footnotes

Other indirect costs associated with DM, such as presenteeism (reduced work productivity while working), could not be estimated due to data unavailability.

## Abbreviations

CIHA: Sistema de Comunicação de Informação Ambulatorial e Hospitalar (Communication System of Hospital and Ambulatory Information)

DM: *Diabetes mellitus*

DRG: Diagnosis Related Group

IBGE: Instituto Brasileiro de Geografia e Estatística (Brazilian Institute of Geography and Statistics)

ICD: International Classification of Diseases

ICU: Intensive Care Unit

IPCA-e: Índice Nacional de Preços ao Consumido Amplo Especial (National Consumer Price Index)

NCDs: Non-communicable diseases

POF: Pesquisa de Orçamentos Familiares (National Household Budget Survey)

SIHSUS: Sistema de Informaço˜es Hospitalares do SUS (National Public Healthcare Hospitalization System)

SUS: Sistema Único de Saúde (National Healthcare System)
