# Supplementary material for: Direct and Indirect Costs of Diabetes in Brazil in 2016
Source: Ann Glob Health. 2022 Mar 3;88(1):14. doi: 10.5334/aogh.3000 (PMC8896241; doi:10.5334/aogh.3000)
Supplement: Appendix A. — Costs attributed to DM – Hospitalization and ambulatory. [file agh-88-1-3000-s1.pdf]

Appendix A - Costs attributed to DM – Hospitalization and ambulatory

The main complications attributed to DM are disaggregated into five groups of diseases: cardiovascular; renal; ophthalmic; neoplasm; and others. The most relevant diseases, in terms of total costs, are cardiovascular diseases due to DM. They accounted for 42.5% (US$ 109 million) of total hospitalization and ambulatory costs. The second most important are renal diseases, which represented 19.7% of the direct costs from diseases related to DM, or US$50.6 million. Ophthalmic diseases are also very relevant, with costs of US$39.8 million (15.5%). Neoplasm diseases due to DM, on the other hand, accounted for 9.5% (US$24.4 million) of those total direct costs. Finally, other diseases represented 12.8% (US$32.8 million) of total hospitalization and ambulatory costs attributed to DM.
