# Supplementary material for: Direct and Indirect Costs of Diabetes in Brazil in 2016
Source: Ann Glob Health. 2022 Mar 3;88(1):14. doi: 10.5334/aogh.3000 (PMC8896241; doi:10.5334/aogh.3000)
Supplement: Appendix B. — Direct Costs – DM and Morbidities. [file agh-88-1-3000-s2.pdf]

Appendix B - Direct Costs - DM and Morbidities

Table 7: Total direct costs using DRG prices for private services, in 2016 US$.

	Diabetes				Morbidities			
	SUS		Non-SUS		SUS		Non-SUS	
	Female	Male	Female	Male	Female	Male	Female	Male
**Hospitalization cost**								
Groups								
below 18 years old	1,839,116.90	1,188,866.06	248,501.30	185,734.81				
18–34 years old	4,870,428.80	1,993,511.07	605,046.69	246,067.96				
35–44 years old	2,695,680.60	2,769,575.10	585,911.13	219,284.76	1,519,300.70	1,747,173.00	235,194.33	281,316.92
45–54 years old	2,964,576.89	3,851,663.76	326,524.08	482,653.05	5,937,992.83	7,996,699.14	893,408.86	1,333,386.49
55–64 years old	4,571,851.72	5,578,708.47	515,400.04	800,715.57	16,742,800.15	24,402,537.45	2,869,079.96	4,670,614.53
65–74 years old	4,876,710.41	4,998,014.04	675,786.12	1,006,577.87	20,975,948.98	28,068,575.63	4,529,913.32	7,072,699.18
75 years old and above	4,511,282.42	3,455,800.05	1,122,727.09	939,295.89	17,120,949.64	15,146,979.69	7,405,410.69	5,713,332.97
Total hospitalizations	26,329,647.74	23,836,138.56	4,086,896.44	3,880,329.91	62,296,992.30	77,361,964.90	15,933,007.16	19,071,350.08
**Ambulatory cost**								
Groups								
below 18 years old	17,955.97	17,962.22	2,850.77	2,977.77				
18–34 years old	71,891.82	65,147.47	8,077.29	10,052.20				
35–44 years old	162,365.00	87,366.18	12,883.31	11,524.28	1,228,194.71	809,162.43	35,567.17	23,688.19
45–54 years old	278,178.88	374,373.72	22,690.77	41,619.61	4,580,599.44	3,316,093.62	138,798.80	96,475.35
55–64 years old	558,638.73	627,134.11	49,432.86	61,203.15	12,771,904.99	9,949,917.66	440,094.42	342,830.44
65–74 years old	386,944.93	449,877.46	42,570.08	50,534.39	17,117,502.	13,078,640.	698,549.31	522,242.21

75 years old and above	259,865.19	122,594.29	40,118.78	21,389.76	79<br>9,561,726.33	43<br>6,608,834.26	501,177.85	306,007.15
Total ambulatory	1,735,840.51	1,744,455.45	178,623.86	199,301.16	45,259,928.25	33,762,648.40	1,814,187.55	1,291,243.34
Popular Pharmacy				304,234,400.00				
POF (material)				10,016,764.00				
**TOTAL**				**633,033,719.62**				

Notes: DRG Brazil collects data from more than 200 hospitals in the country and 4,909 patients hospitalized due to DM or complications from the disease (2016).

Table 8: Total indirect costs using DRG prices for private services, in 2016 US$.

	Diabetes		Morbidities	
	Female	Male	Female	Male
**Absenteeism cost**				
Groups				
below 18 years old	580,508.81	425,327.28		
18–34 years old	1,343,757.75	496,369.97		
35–44 years old	988,542.75	731,137.94	192,166.86	225,476.36
45–54 years old	1,216,245.63	1,662,624.50	733,241.75	938,137.88
55–64 years old	1,928,707.50	2,504,171.75	2,045,643.75	2,843,334.75
65–74 years old	2,089,243.75	2,233,472.25	2,876,845.50	3,661,182.50
75 years old and above	2,052,454.63	1,535,072.00	3,675,572.75	2,817,755.25
Total absenteeism	10,199,460.81	9,588,175.69	9,523,470.61	10,485,886.73
**Early death cost**				
Groups				
18–34 years old	82,067,216.67	88,152,875.19		
35–44 years old	103,376,179.91	134,543,304.55		
45–54 years old	167,510,805.92	288,475,875.30		
55–64 years old	55,849,680.96	263,518,382.08		
Total early death	408,803,883.47	774,690,437.12		
INSS	186,363,308.25	110,364,389.00		
**TOTAL**		**1,520,019,011.69**		

Notes: DRG Brazil collects data from more than 200 hospitals in the country and 4,909 patients hospitalized due to DM or complications from the disease (2016).

	**SUS**	**non-SUS**	**Total**
Diabetes	137,094	13,377	150,471
Attributed to DM	124,588	24,551	149,139
Total	261,682	37,928	299,610

Table 9: Economic burden of DM per patient and *per capita*, in 2016 values.
